# Household size is critical to *varicella-zoster* virus transmission in the tropics despite lower viral infectivity

**DOI:** 10.1016/j.epidem.2010.11.003

**Published:** 2011-03

**Authors:** Richard A. Nichols, Karin T. Averbeck, Anja G. Poulsen, Mahmoud M. al Bassam, Fernando Cabral, Peter Aaby, Judith Breuer

**Affiliations:** a School of Biological and Chemical Sciences, Queen Mary University of London, London E1 4NS, UK; b Division of Infection & Immunity, University College London, 46 Cleveland Street, London W1T 4JF, UK; c Bandim Health Project, Statens Serum Institut, Apartado 861, Bissau, SAB, Guinea-Bissau

**Keywords:** Chickenpox, VZV, Climate, Tropical, Transmission

## Abstract

The epidemiology and severity of infections can vary dramatically in different geographical regions. Varicella zoster virus (VZV) is a particularly tractable model for investigating such global differences, since infections can be unambiguously identified. VZV is spread by aerosol to cause chickenpox, which, in temperate countries, is a relatively benign childhood infection; yet in tropical countries it tends to occur at later age, a trend associated with markedly increased severity including complications, hospitalization, and overall burden of care.

To investigate global differences in the epidemiology of chickenpox we studied a population in Guinea Bissau, which in contrast to other tropical countries has an unexpectedly early age of infection with VZV, comparable to temperate latitudes. In this study we used detailed records from over 3000 houses during an outbreak of chickenpox, combined with viral genetic information on routes of infection, to obtain precise estimates of disease transmission within and between houses. This community contains many large households in which different families live under a single roof, in living quarters divided by partitions. Our data show that household infectivity in tropical Guinea Bissau is reduced four-fold compared with temperate climates (14.8% versus 61–85%), with an intermediate rate between members of the same family who are in more intimate contact (23.5%). All else being equal, these lower infection rates would be expected to lead to a later age of infection as is commonly seen in other tropical countries. The young age of infection, which had drawn our attention to the Guinea Bissau population, can however be explained by the exceptionally large household sizes (mean 14.5 people).

We have combined genetic and demographic data to show that the epidemiology of chickenpox in tropical Guinea Bissau is dependent on the interaction of the social and physical environments. The distinctive clinical presentation of VZV and its ubiquitous distribution make it an attractive model for estimating the variables that contribute to global differences in the transmission of airborne viruses.

## Introduction

The severity and epidemiology of diseases can vary from place to place, but the underlying causes of such differences remain understudied (Chowell et al., 2008). Understanding such global differences in disease becomes pressing, as development transforms human demography and mobility causing key changes to transmission patterns (Hollingsworth et al., 2007), and to the environment in which the disease propagates.

Infection by varicella zoster virus (VZV) is a particularly tractable model for studying global differences in disease severity, since infectious are readily and unambiguously identified and there are considerable differences between localities. In developed temperate countries VZV infection causes a relatively benign childhood infection (varicella or chickenpox). However, in many tropical countries infection tends to occur at later age (Marin et al., 2008), a trend associated with markedly increased severity including complications such as pneumonia, hospitalization, and a greater overall burden of care (Almuneef et al., 2006). The timing of infection is reflected in age-adjusted seroprevalence, which shows correlations suggesting a later age of infection in warmer more rural regions (Lolekha et al., 2001). In this paper we attempt to dissect out the causes of such correlations by obtaining direct estimates of infection rates and by combining epidemiological records with genetic data to verify transmission routes.

The study focused on the unexpected epidemiology in Guinea Bissau. Unlike other tropical countries such as Thailand (Lolekha et al., 2001), India (Lee, 1998; Lokeshwar et al., 2000), Singapore (Dashraath et al., 2007), and the West Indies (Garnett et al., 1993), Guinea Bissau has a predominantly childhood onset of chickenpox. The median age of infection is 4 years (Poulsen et al., 2005), and by 10 years 90% of the population is infected, estimated from attack rates in this study. This pattern resembles the epidemiology in temperate countries, such as the UK and the USA before the introduction of vaccine, where the severity of disease is correspondingly mild (Ross, 1962; Simpson, 1952).

We speculated that the earlier age of infection in Guinea Bissau might be explained by a higher infectivity — comparable to that in temperate climates, due to genetic differences in the virus or its environment. The synthesis of genetic and epidemiological data produced precise estimates of infectivity, which were built into a model that predicted the effects on age of infection and hence disease severity.

## Methods

Samples and data were collected in collaboration with Bandim Health Project in Bissau, as part of an epidemiological study including 44,034 individuals in 3068 households and recording demographic and clinical data for 1446 VZV cases (Poulsen et al., 2005). The study gained ethics approval from the Local Research Ethics Committee and of the East London and City Health Authority. Houses typically comprised 3–4 households (families) living under the same roof with a shared indoor living space in which rooms have no ceiling (Poulsen et al., 2005). During a single outbreak (Nov 2000–June 2001) 400 VZV samples for genotyping were collected from patients with chickenpox. The very large sample size and detailed records allowed the calculation of the rate of infection between houses, from observations of primary infections. The genetic data allowed secondary, and subsequent infections within a house to be distinguished from new inter-house infections, and hence the rate of within house infection could also be calculated.

### Viral genotyping

SNP variation was scored in the origin of replication (Muir et al., 2002) and the classification into clades (the broad classification of VZV viral genotypes) confirmed by SNP analysis of genes 1 and 21, 22, 50 and 54 (Muir et al., 2002; Peters et al., 2006; Norberg et al., 2006). Of the 400 cases, 394 had genotypes from Clade 5, commonly found in Asia and Africa. Clade 1, prevalent in European-derived populations, accounted for the remaining six cases, which occurred in two separate houses, both following the return of a child with chickenpox from abroad. The hypervariable region of the origin of replication (OriS) was genotyped to distinguish different viruses within the outbreak. The genotypes were recorded as the number of [TA]_N_ and [GA]_N_ repeats in OriS.

### Estimating the parameters describing viral transmission

The analysis was carried out using the statistical package R, and the code is appended as supplementary material in Appendix A. The statistical model made use of the fact that individuals become infectious when skin lesions appear. There will, however, be a delay before the detection of transmission to another individual in the same house, because infection is not instantaneous and due to the incubation period of the disease. The probability of the observations of subsequent infections was therefore modeled as a function of *d,* the number of days since lesions were observed in the infectious individual:

(1) λ(d|g,m,σ,β)=Γ(d−g,m,σ)β.

This equation describes the rise and fall of infectiousness using the gamma probability density Γ(), with mean *m* and variance *σ*. The parameter *g* specifies the period of delay before subsequent infections (the latent period between infection and the appearance of lesions). Infectivity is given by the proportion, *β,* of susceptible individuals who become infected by the initial case. The notation Λ*(d*| *g, m, σ, β*) is used for the upper tail of the corresponding cumulative probability.

The analysis also had to model primary infections: those from outside the house. The frequency of such infections changed throughout the outbreak, and was estimated by first identifying, for each day, houses with no infectious individuals in the preceding period (7–25 days). Any infections in these houses can therefore be assumed to have come from outside. The total number of susceptible individuals on day *t* of the outbreak *N*_*su*_*(t)* in these uninfected houses was estimated from the ages of their inhabitants and the household size as

$$N_{\mathrm{su}}\left(t\right)=\sum s(a_{\text{it}},n_{\text{i}})\text{.}$$

**Fig. 1:**
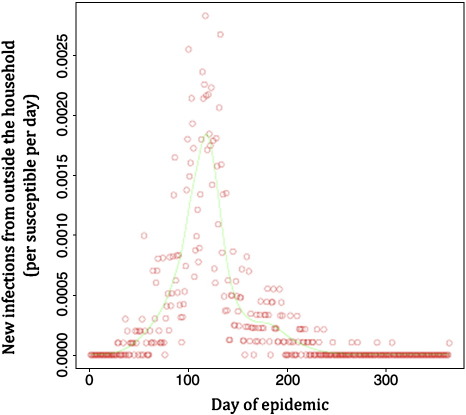
Rate of primary infections in a household. Rates were calculated per susceptible individual per day. Observed data (circles) and fitted data (line) are shown.

The function *s(a*_it_,*n*_i_) was estimated from the ages of infected individuals as set out in the next section. The number of individuals who became infected in these households on each day, *x*_*u*_*(t)* had been observed, so the rate of infection on each day could be estimated as *x*_*u*_*(t)/N*_*su*_*(t)*. These values are plotted in Fig. 1, along with the trend fitted by a generalized additive model using the ‘gam’ function in R. These fitted values are the estimates of the daily rate of primary infection on day *t* of the outbreak, *Β(t).*

These estimates were combined with the rate of within-household infections as follows. For each individual, *i,* in the survey, we have records of

*a*_*i*_: their age at the start of the outbreak;
*φ*_*i*_: whether they were infected during the outbreak (*φ = 1*) or not (*φ = 0*);
*ϑ*_*i*_: the day of the outbreak on which they were infected (if any);
*d*_*itk*_: the number of days that had passed on day *t* of the outbreak since housemate, *k,* became infectious.

The probability of a susceptible individual becoming infected can be expressed in terms of the cumulative distribution function C*(t,i)* that they remained uninfected from either outside the house or by housemates until day, *t*:

(2) $$\text{C}(t,i)=\prod_{m=1}^{t}\left(1-\text{B}(m)\right)\prod_{k}\Lambda \left(d_{\mathrm{imk}}|g,m\sigma ,\beta_{\mathrm{ik}}\right)\text{.}$$

$\prod_{m=1}^{t}\left(1-\text{B}(m)\right)$ is the probability that the individual escapes primary infection from outside the house until day *t,* and Λ*(d*_*itk*_| *g, m, σ, β*_*ik*_) is the probability of escaping infection by housemate *k,* who has been infectious for *d*_*itk*_ days on day *t* (*c.f.* Eq. (1))*.* The notation *β*_*ik*_ indicates that the rate can vary depending on the level of contact between individuals *i* and *k,* living in the same house*.* Since the individuals in the same family unit may be in more intimate contact, two different values for *β*_*ik*_ were estimated one for transmission between members of the same family, *β*_*f*_; the other for the same household but not the same family, *β*_*h*_. We also explored the possibility that contact between members of different families might be lower in larger households, so that the rate is a function of the household size, *n*:

(3) $$\beta_{\mathrm{hn}}={\mathrm{logit}}^{-1}\left(m\Delta n+\mathrm{logit}(\beta_{h})\right)\text{.}$$

The logit transform ensures that the rate does not exceed 1. The parameter *β*_*h*_ is the rate for the largest household size and *Δn* deviation from maximum household size.

The probability of being infected on day *ϑ*_*i*_ is given by the difference of the value given by equation at the start and end of day *ϑ*_*i*_, multiplied by probability of being susceptible at the start of the outbreak (given the individual's age and household size), *s(a*_*i*_*,*n_i_*)*:

(4) $$s(a_{i},n_{i})\left[C(\vartheta_{i},i)-C(\vartheta_{i}+1,i)\right]\text{.}$$

The probability of those observations in which the individual remained uninfected throughout the infection is given by the probability of being susceptible at the start of the outbreak and escaping infection for the whole 364 day outbreak (C(364,i)) plus the probability of having been previously infected, before the start of the outbreak:

(5) $$\text{s}(a_{i},n_{i})\text{C}(364,i)+1-\text{s}(a_{i},n_{i})\text{.}$$

The likelihood of the observations also included the information on viral genotype. The calculation took into account the changing frequency of genotypes during the course of the outbreak. The frequency, *p*_*jt*_, of genotype *j* on day *t* was estimated by regressing the multinomial frequencies against time using the multinom function from the nnet R package. For any genotype *j* it was also necessary to calculate the frequency of the *m* genotypes one mutational step away from *j*, $p_{\mathrm{j\Sigma t}}=\sum_{m}p_{\mathrm{jmt}}\text{.}$

The genetic data could be included in the likelihood in those cases where the all the infectious individuals in the house had been genotyped (or were primary infections). For each infectious individual, *k*, the genotype data can be represented by an indicator variable *I*_*ikn*_ {*n* ∈ 0,1,*u*) where 0 indicates that there are no differences between the genotype of the infectious individual and the new infection, *i* (an exact match between the genotypes of *i* and *k*), 1 indicates the genotypes differ by a single mutational step, and *u* indicates that the infectious individual was an untyped primary infection.

In the case of an infection from an untyped individual (*i.e.* from outside the house, or an untyped primary infection within the house) the probability of the genotype is give by *(1 − μ) p*_*jt*_ + *μp*_*jΣt*_*,* where *μ* is the mutation rate. Similarly if the genotype of the infectious individual is known, the probability of the observed genotype is

$$(1-\mu )I_{ik0}+\mu I_{ik1}\text{.}$$

The parameter *γ*_*ip*_ quantifies the relative probability that newly infected individual, *i*, was a primary infection, and *γ*_*ik*_ the relative probability of infection by each of the k infectious individuals in the same household (i.e. *γ*_*ip*_ + Σ*γ*_*ik*_ = 1). The values depend on the day of infection *ϑ*_*i*_ and parameters *g, m, σ*, *β*_*m*_ and *β*_*f*_ as follows:

(6) $$\begin{array}{l} \gamma_{\mathrm{ip}}\propto \prod_{m=1}^{\vartheta_{i}}\left(1-\text{B(}m\text{)}\right)-\prod_{m=1}^{\vartheta_{i}+1}\left(1-\text{B(}m\text{)}\right),\\ \gamma_{\mathrm{ik}}\propto \Lambda (d_{i\vartheta_{i}k}|g,m,\sigma ,\beta_{\mathrm{ik}})-\Lambda (d_{i(\vartheta_{i}+1)k}|g,m,\sigma ,\beta_{\mathrm{ik}}) \end{array}$$

Hence the probability that the genotype of newly infected individual, *i*, has the observed genotype, *j* is

(7) $$\begin{array}{l} \pi_{j}=\gamma_{\mathrm{ip}}\left[\mu p_{j\vartheta_{i}}+(1-\mu )p_{j\Sigma \vartheta_{i}}\right]+\sum_{k}\gamma_{\mathrm{ik}}\left(I_{\mathrm{iku}}\left[\mu p_{j\vartheta_{i}}+(1-\mu )p_{j\Sigma \vartheta_{i}}\right]+\mu I_{ik1}+(1-\mu )I_{ik0}\right);\\ \text{or}\;\pi_{j}=1\text{,} \end{array}$$

when no informative genotype was obtained*.*

Combining Eqs. (3), (4) and (6) the likelihood of the data was calculated as

(8) $$\prod_{i}\varphi_{i}s(a_{i},n_{i})\left[\text{C}(\vartheta_{i},i)-\text{C}(\vartheta_{i}+1,i)\right]\pi_{j}+(1-\varphi_{i})\left[s(a_{i},n_{i})\text{C}(364,i)+\left(1-(a_{i},n_{i})\right)\right]\text{.}$$

**Fig. 2:**
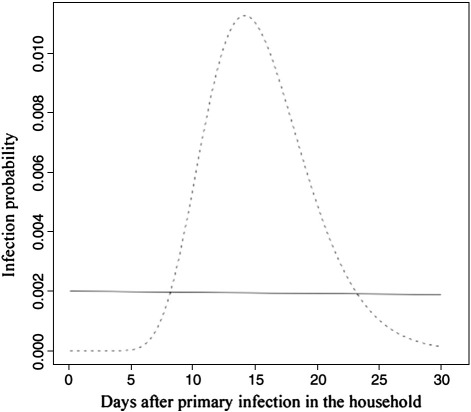
The probability of infection (of a susceptible co-habitant). The dashed curve shows the maximum likelihood estimate of the change in the probability with time since the initial case. For comparison the solid lines shows the probability of becoming infected from outside the household at the peak of the outbreak. The total area under the dashed curve is the infectivity: only 0.125 of susceptible individuals are infected by each primary case.

Note that the dependence on parameters *g, m, σ*, *β*_*f*_, *β*_*h*_*, m* and *μ* has been suppressed in this notation, but is explicit in Eqs. (2), (3), (6) and (7). Eq. (8) combines the epidemiological data with the genetic data in a single likelihood. The maximum likelihood values and standard errors of these parameters were estimated using the mle function in R. Five of the parameters specify the curve (shown in Fig. 2) describing the rate of within-house infection: *m,σ, g, β*_*f*_ and *β*_*h*_; in addition, the mutation rate is *μ,* and *m* quantifies any decline in infectivity with household size.

### Estimating and evaluating the distribution of susceptible individuals

The function to estimate the distribution of susceptible individuals made use of the data on the age of infection, which showed a slight but significant association with the size of the household (e.g. the mean age of infection was 5.51 for households containing fewer than 15 individuals, and 5.11 for those containing over 35). This trend was estimated using by finding the maximum likelihood values of the four parameters of the following probability distribution for the age of infection, as a function of age in days, *d,* and household size, *n*:

$$\text{P}\left(d,n\right)=\text{G}(d,m_{n},s_{n})\text{,}$$

in which the mean and variance are functions of the household size: *m*_*n*_ *= k*_*n*_*θ*_*n*_*; σ*_*n*_ *= k*_*n*_*θ*_*n*_^*2*^. The parameters *k* and *θ* are conventionally described as the shape and the scale of the distribution, respectively. They were estimated as linear functions of *n*:

$$k_{n}=\text{a}+bn\text{,}\;\text{and}\;\theta_{n}=c+dn\text{.}$$

The maximum likelihood values of *a, b, c* and *d* were estimated from the vector of infection ages and corresponding household sizes using the MLE function of R. Since the distribution of ages was essentially uniform over the ages of infection (up to 30 years), the cumulative gamma distribution with the fitted values (a = 1.09, b = 0.00880, c = 2061, d = −2.07) provides an estimate of *s(a,n).* The estimates were robust to the use of different datasets: restricting the data to infections known to be from outside the household led produce no significant difference in the parameter estimates (all were within 1 SE) and the effect on mean value of *s(a,n)* was minimal: decreasing it by less than 0.07%. Another issue is that households could differ in the age-distribution of susceptible individuals. For example households infected in recent outbreaks might have a younger average age of infection, and not be infected in the latest outbreak. A strong effect appeared unlikely, given the low estimates of infectivity (below). However, we examined the robustness of our estimates by substantially reducing our estimates of the number of susceptible individuals in those households that were not infected in that latest outbreak. The values of s(a*,n*) were transformed to s′(a*,n*) = logit^−1^(0.5 + logit (s(a*,n*))), which reduced the median age of infection by 1.2 years. The analysis was repeated and had negligible effect on most parameters, and a reduction of less than 3% for the parameters of major biological interest *β*_*f*_ and *β*_*h.*_

### Modeling the effect of different household sizes and infection parameters

The model was specified in language R, and is provided in a supplementary file in Appendix A. In outline, we knew the number and date of birth of the individuals in each house. We could therefore obtain empirical estimates of probability of observing a specified number of births (which ranged from 0 to 6 in the census year) as a function of inhabitants per house. We also obtained estimates of the number of years since a previous birth as a function of inhabitants per house.

These values allowed us to construct a model, which we initiated with households with the observed size distribution, and comprising entirely of susceptible individuals. We then initiated infections from outside each house at annual rate *r* (obtained from the above analysis) and subsequent internal secondary and tertiary infections as a Poisson process with rates of *β*_*f*_ and *β*_*h* —_ assuming the proportion,*ϕ*, of intra family contacts declined with household size, following the observed inverse relationships in the real data (*ϕ =* 1.74*/n* + 0.318). New susceptibles were then introduced each year at the empirically observed birth rates and the entire process iterated until the proportion of infected individuals stabilized. At each iteration, the proportion of houses with every possible number of infections was recorded (in the range 0 to the number of inhabitants). Separate records were kept for each number of years since the last birth (up to a maximum of 40 years), since that affected the number of susceptible individuals. The distribution of infected individuals, in houses in which there were new births, was determined using the empirical estimates of the gaps between births to produce a weighted average for each household size. The expected distribution of the age of infection was obtained by following notional susceptible individuals introduced, to houses of each size, over subsequent rounds of infection and birth. The model was then re-run with the inhabitants per house corresponding to single family households (in which the household size before birth had mean 5, and the corresponding Poisson distribution truncated at 2 and 10). The procedure was repeated with a household infection rate multiplied by 2.6*,* matching the values reported for single-family households in temperate climates (0.6–0.85).

## Results

Primary infections were defined as those occurring during a period when the house had not contained an infectious individual in the preceding 7–28 days. There was no significant difference in the age of primary and secondary cases (mean ages 5.6 (*N* = 999) and 5.3 years (*N* = 461), respectively; *t*-test *P* = 0.54). The rate of primary infections, and an estimate of the number of susceptible individuals in these houses (see methods) were used to calculate the rate of between-house infection. This value rose and fell as the epidemic proceeded (Fig. 1). At the peak of the outbreak, the estimated primary infection rate averaged 16 new household infections per 10,000 susceptible individuals in uninfected households per day; corresponding closely to the observed rate of 15 infections which had been putatively classified as “primary” by the survey team.

**Fig. 3:**
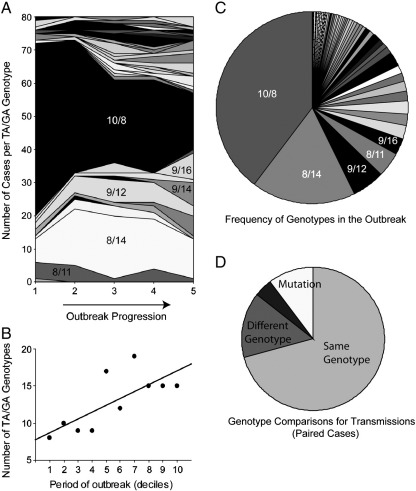
High levels of variation in the VZV origin of DNA replication TA and GA repeats during a single outbreak. A) Change in genotype frequencies over time. Each shade represents a different combination of number of TA and GA repeats. Two numbers are used to describe the number of repeats in the common variants: for example, 10/8 specifies 10 TA repeats followed by 8 GA repeats. Each outbreak period indicated on the x-axis represents 80 cases. B) A graph illustrating the increasing number of genotypes circulating as the outbreak proceeds. The outbreak is divided into 10 periods, each comprising 40 cases. C) Genotype frequencies (averaged over the whole outbreak). D) A comparison of the genotypes obtained from pairs cases occurring within the same household, within the expected incubation period. If the latter case were due to the virus being transmitted from the first, the genotypes would be expected to be identical or to differ because of mutation. For only 67% of such paired cases were the genotypes identical. For 12%, the genotypes differed by one repeat of either TA or GA, suggesting a replication slippage mutation event. In 21% of cases the genotypes differed by two or more, suggesting that the second infection was not actually due to viral transmission from the first.

The genetic data were used to infer the frequency of secondary infections arising from these primary cases (and subsequent infections). The genotypes are recorded as the number of [TA]_N_ and [GA]_N_ repeats in the hypervariable region of the origin of replication (OriS), which is sufficiently polymorphic to distinguish different viruses within the outbreak Forty-nine TA/GA genotypes were identified, with increasing diversity over the course of the outbreak (Fig. 3). The full analysis combined the genetic and epidemiological data (see Eq. (7)), but some important principles can be established straightforwardly by inspection of that subset of the data for which all possible infectious individuals within a house had been genotyped. Firstly, in some (5, 12%) cases that appeared to be secondary infections, the genotype differed by one repeat (in either the TA or GA array) from that of the primary infection (Fig. 3D). These cases could either be mutations, or infections from outside the house. Those alternatives had to be taken into account in the full analysis. More important, there were a number of other cases (9, 21%) in which the genotypes differed by at least two repeat units, and were hence almost certainly infections from outside the house. On the other hand, even when both genotypes matched, it remained possible that the infection had come from outside the house.

In order to take these possibilities into account, we used the full genetic dataset to estimate the frequency of each genotype throughout the outbreak. Our estimates of the rate of between-house infections were used, together with the genotype data and timing of subsequent infections in a house, to model the rise and fall of within-house transmission rates after the primary infections.

We obtained maximum likelihood estimates and confidence intervals for the five parameters. The corresponding curve for infection rate, as a function of time, is illustrated by the dashed curve in Fig. 2. This distribution indicates that 95% of within-house transmissions occurred between days 9.3–24.1, as opposed to 10–29 days previously estimated from the same outbreak, but without knowledge of the genetic data (Poulsen et al., 2005), and consistent with estimates of 10–23 days incubation from direct observations of home-dwelling children (Dashraath et al., 2007) and 8–21 days from another family-based study (Simpson, 1952). This rate is compared to the rate of infection from outside the house at the height of the infection (0.02 per susceptible per day, solid curve in Fig. 2). The OriS mutation rate, *μ*, was estimated as 0.161 (SE 0.067) per transmission, close to the raw estimate from the subset of the data (0.12). Most importantly for the interpretation of the data, it is estimated that only 23.5% (SE 2.2%) susceptible individuals within the same family were infected by each index infection, and 14.8% (SE 1.0%) of susceptible individuals in other families (in the same house).

**Fig. 4:**
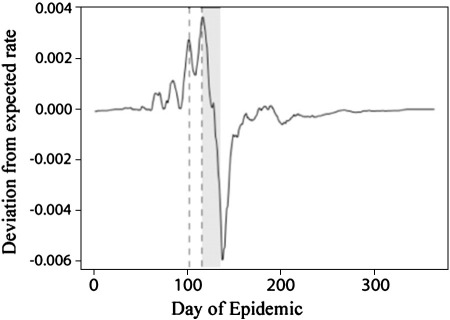
Between-household infection efficiency. The curve shows the deviation from expected rate of new infections (based on the regression on the number in the preceding infectious period). Vertical dashed lines show the beginning and end of the school summer break, and the gray area is the same period offset by the mean lag time for viral incubation shown in Fig. 1. The sharp drop in infection rates within this shaded region corresponding to the school break. The onset of the rains much later (c. day 200).

Having obtained a more precise estimate of the infectious period it is possible to re-plot the graph of primary infections to illustrate changes in transmission efficiency. Fig. 4 shows the deviation from the regression of primary infection rate on the number of infectious individuals (in the corresponding infectious period). Plotted on the same time scale is the period of the spring school holiday, offset by the mean infectious period to take into account that infections contracted in the school holiday would not be detected until lesions appear.

**Fig. 5:**
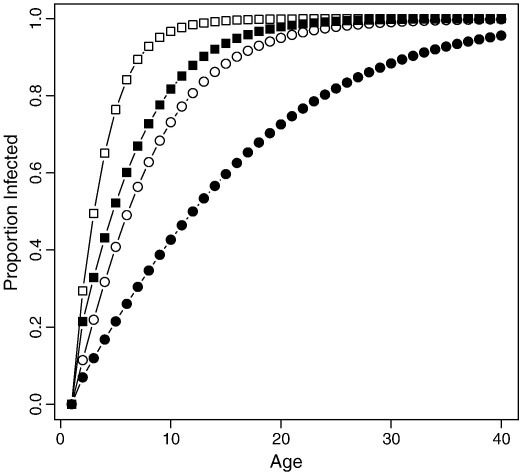
The expected distribution of the age of infection. The two curves with circular symbols correspond to the simulations with the observed rate of household infections in Guinea Bissau (*β*_*f*_ *=* 0.235 l; *β*_*h*_ = 0.148). Two curves with square symbols correspond to simulations with infection rates observed in temperate climates (2.6 times higher). In each pair the open symbols show the results for the observed distribution of household sizes in Guinea Bissau (mean 14 individuals), and the closed symbols show the results for small households (mean 5). Hence the shallower curve with filled circles indicates that, had the household size been smaller, the low infection rate in Guinea Bissau would have resulted in a later average age of infection, corresponding with other tropical countries. The two central curves show similar early ages of infection as observed in both Guinea Bissau and most temperate countries. The steepest curve is expected in larger households in temperate climates. Comparable data are reported from the Netherlands where mixing in large nursery classes occurs at an early age (Nardone et al., 2007).

The model constructed to investigate the effect of the observed parameter values on the age of infection generated Fig. 5. The curves show the expected cumulative frequency for the age of infection — which predicts the age specific seroprevalence. The two central distributions are similar (filled squares and open circles) but can be explained by lower infectivity and higher household sizes (the parameter estimates estimated in Guinea Bissau) or by higher infectivity (as estimated in some temperate climates (12)) and smaller household size.

## Discussion

The population in Guinea Bissau had drawn the attention of this study because, unlike other tropical localities, it showed an early age of varicella infection: an age distribution comparable to that seen in temperate countries such as those of northern Europe. The viral strains were not typical of Europe. The SNP data show that there was only a very low frequency of viral strains from a clade typically found in Europe; these were only found in two households with children who had recently traveled overseas, and we found no evidence that they had propagated beyond the household. The remainder of the genotypes was from clades also found in other tropical countries. The early pattern of infection could have been explained if, for genetic or environmental reasons, the infectivity of the virus was high. However, our estimates show a robust and substantial trend in quite the opposite direction: a four-fold reduction in infectivity to only 23.5% (SE 2.2%) of susceptible individuals in the same family compared with estimates from temperate climates of 61 – 85% (Ross, 1962; Simpson, 1952). Members of different families within the same house live in very close proximity (Poulsen et al., 2005): the living quarters are divided by partitions, which typically do not reach the roof. Given that the virus is spread by aerosol, and the communal areas within each house, it is striking that the estimated transmission is significantly lower (14.9%; SE 1.0%). The reduced infectivity in Guinea Bissau, and the elevated rate only amongst those in the most intimate contact consistent with *in vitro* evidence that VZV infectivity and that of other airborne viruses, is reduced by heat, UV light and humidity (Lytle and Sagripanti, 2005), all of which are higher in tropical than in temperate regions. Similarly, loss of envelope lipid ordering, which is crucial for stability and airborne transmission of Influenza A virus, has recently been reported to occur at temperatures higher than physiological levels (Polozov et al., 2008). Evidence that airborne viral transmission is indeed lower at higher temperatures has been reported in a guinea pig model (Lowen et al., 2008).

All else being equal this low rate would lead to a later age of infection with an associated increase in disease severity. Higher rates of infectivity were detected in the smaller households, but these could be attributed to the greater proportion of intra-family contacts (since smaller households necessarily contain a smaller number of family units — the proportion of interfamily contacts had a inverse relationship with household size, see Methods). Once this effect is taken into account, there was no significant effect of household size on infectivity: the estimate of *m* = −0.0065, SE 0.0072.

The data also show a large number of inhabitants per house (on average 3.5 families or 14 individuals), which the key to understanding the early age of infection despite the low infectivity. The more inhabitants the greater the birth rate, consequently the average size for a newborn's house is even larger containing 24 individuals, with on average 0.97 births per year. The model (Fig. 5). Shows that the large number of in inhabitants per house in Guinea Bissau is indeed sufficient to explain the early age of infection, comparable to a temperate country, even given the observed within-house rate of infection nearly five times lower. Conversely the later age of infection in other tropical countries can be explained by the smaller household sizes (e.g. 4.7–5.5 in Thailand, India and Singapore), although there are other possible explanations: the transmission rate could also be affected by other influences on intimate contact rates such as the sequestering of infected individuals (Garnett et al., 1993).

The estimates shown in Figs. 1 and 4 suggest that the epidemic spread of the virus was highly sensitive to transmission between houses. Indeed the decline of the outbreak coincides with the beginning of the spring school holidays. This period did not correspond to a dramatic change in climate as has been reported for measles (Ferrari et al., 2008), being 80 days before the onset of the rainy season (day 200 in Fig. 4). Rather the timing suggests that the persistence of this epidemic requires the mixing of contacts between houses that occurs at school. A similar effect has been suggested for influenza infections (Cauchemez et al., 2008).

Benchmark studies of within-household transmission in temperate climates range from 61% in a rural English town to 87% in a New York City suburb, with median ages of infection similar to those found in Bissau (Ross, 1962; Simpson, 1952). In both UK and US analyses, primary cases were significantly older than secondary cases, as a school-aged child (ages 5–9) tended to contract the virus outside the house, bring it home, and subsequently infect others, including the non-school-aged children (ages 0–4), within the house (Ross, 1962; Simpson, 1952). In contrast, in Guinea Bissau, we found no significant difference in the age of primary and secondary cases, which may be explained by preschool-aged children being exposed to infection by older children in the large houses, the extensive carriage of children on the backs of their mothers and the constant outdoor mixing of pre-school children in this hot climate (Aaby, personal communication).

Others have modeled the spread of VZV, using time-of-exposure matrices to capture the mixing between different individuals (Zagheni et al., 2008) and how they change through time (Whitaker and Farrington, 2004). This study has been able to extend this approach by obtaining direct estimates of transmission within an epidemic outbreak. We were able to distinguish rates within and between houses, and between families within houses. Two aspects of VZV biology allowed accurate estimation. First, clinical diagnosis of the infection, unlike many other respiratory viral infections, is almost completely unambiguous (since the eradication of smallpox) and does not require laboratory confirmation. Secondly, it was possible to use a highly polymorphic region of the viral genotype to distinguish the different sources of viral infections.

The more general implications extend beyond the VZV system. The results highlight the importance of the clustering of susceptible individuals into households or similar environments including preschool kindergartens in temperate Netherlands (Nardone et al., 2007), rather than population density *per se* in determining the age of infection. We predict that similar studies in those regions with later disease-onset would reveal a similarly low infectivity but smaller household sizes. In Guinea Bissau, public health policy changes or other events which impact the number of people living in the same house would profoundly affect the epidemiology of VZV and increase its disease burden. Significant change to a more tropical climate could have the same effect in temperate countries, although the widespread introduction of infant vaccination in these countries is likely to counter this trend.

## References

[bb0005] Almuneef M., Memish Z., Balkhy H., Alotaibi B., Helmy M. (2006). Chickenpox complications in Saudi Arabia: is it time for routine varicella vaccination?. Int. J. Infect. Dis..

[bb0010] Cauchemez S., Valleron A., Boëlle P., Flahault A., Ferguson N. (2008). Estimating the impact of school closure on influenza transmission from Sentinel data. Nature.

[bb0015] Chowell G., Bettencourt L.M.A., Johnson N., Alonso W.J., Viboud C. (2008). The 1918–1919 influenza pandemic in England and Wales: spatial patterns in transmissibility and mortality impact. Proc. R. Soc. B Biol. Sci..

[bb0020] Dashraath P., Ong E.S., Lee V.J. (2007). Seroepidemiology of varicella and the reliability of a self-reported history of varicella infection in Singapore military recruits. Ann. Acad. Med. Singapore.

[bb0025] Ferrari M., Grais R., Bharti N., Conlan A., Bjørnstad O. (2008). The dynamics of measles in sub-Saharan Africa. Nature.

[bb0030] Garnett G.P., Cox M.J., Bundy D.A.P., Didier J.M., Stcatharine J. (1993). The age of infection with varicella-zoster virus in St-Lucia, West-Indies. Epidemiol. Infect..

[bb0035] Hollingsworth T.D., Ferguson N.M., Anderson R.M. (2007). Frequent travelers and rate of spread of epidemics. Emerg. Infect. Dis..

[bb0040] Lee B.W. (1998). Review of varicella zoster seroepidemiology in India and South-east Asia. Trop. Med. Int. Health.

[bb0045] Lokeshwar M.R., Agrawal A., Subbarao S.D., Chakraborty M.S., Ram Prasad A.V. (2000). Age related seroprevalence of antibodies to varicella in India. Indian Pediatr..

[bb0050] Lolekha S., Tanthiphabha W., Sornchai P., Kosuwan P., Sutra S. (2001). Effect of climatic factors and population density on varicella zoster virus epidemiology within a tropical country. Am. J. Trop. Med. Hyg..

[bb0055] Lowen A., Steel J., Mubareka S., Palese P. (2008). High temperature (30 degrees C) blocks aerosol but not contact transmission of influenza virus. J. Virol..

[bb0060] Lytle C., Sagripanti J. (2005). Predicted inactivation of viruses of relevance to biodefense by solar radiation. J. Virol..

[bb0065] Marin M., Watson T.L., Chaves S.S., Civen R., Watson B.M. (2008). Varicella among adults: data from an active surveillance project, 1995–2005. J. Infect. Dis..

[bb0070] Muir W., Nichols R., Breuer J. (2002). Phylogenetic analysis of varicella-zoster virus: evidence of intercontinental spread of genotypes and recombination. J. Virol..

[bb0075] Nardone A., de Ory F., Carton M., Cohen D., van Damme P. (2007). The comparative sero-epidemiology of varicella zoster virus in 11 countries in the European region. Vaccine.

[bb0080] Norberg P., Liljeqvist J., Bergström T., Sammons S., Schmid D. (2006). Complete-genome phylogenetic approach to varicella-zoster virus evolution: genetic divergence and evidence for recombination. J. Virol..

[bb0085] Peters G., Tyler S., Grose C., Severini A., Gray M. (2006). A full-genome phylogenetic analysis of varicella-zoster virus reveals a novel origin of replication-based genotyping scheme and evidence of recombination between major circulating clades. J. Virol..

[bb0090] Polozov I., Bezrukov L., Gawrisch K., Zimmerberg J. (2008). Progressive ordering with decreasing temperature of the phospholipids of influenza virus. Nat. Chem. Biol..

[bb0095] Poulsen A., Cabral F., Nielsen J., Roth A., Lisse I.M. (2005). Varicella zoster in Guinea-Bissau — intensity of exposure and severity of infection. Pediatr. Infect. Dis. J..

[bb0100] Ross A. (1962). Modification of chicken pox in family contacts by administration of gamma globulin. N. Engl. J. Med..

[bb0105] Simpson R. (1952). Infectiousness of communicable diseases in the household (measles, chickenpox, and mumps). Lancet.

[bb0110] Whitaker H., Farrington C. (2004). Infections with varying contact rates: application to varicella. Biometrics.

[bb0115] Zagheni E., Billari F., Manfredi P., Melegaro A., Mossong J. (2008). Using time-use data to parameterize models for the spread of close-contact infectious diseases. Am. J. Epidemiol..

